# Cancer-related deaths among cases referred for forensic evaluation in Türkiye: a 5-year retrospective analysis

**DOI:** 10.1590/1806-9282.20260577

**Published:** 2026-10-05

**Authors:** Ahmet Burak Aydemir, Veysel Osman Soğukpınar, Humman Şen, Ferah Karayel

**Affiliations:** 1 Ministry of Justice, Council of Forensic Medicine, The Group Presidency of Forensic Medicine – Van, Türkiye.; 2 Ministry of Justice, The Council of Forensic Medicine – Istanbul, Türkiye.

**Keywords:** Autopsy, Neoplasms, Cause of death, Malpractice, Neoplasm metastasis

## Abstract

**OBJECTIVE:**

Cancer is a major global cause of mortality and holds particular importance in forensic cases regarding diagnosis, treatment processes, and determination of cause of death.

**METHODS:**

This retrospective study analyzed 163 cancer-related death cases referred for forensic evaluation to the First Specialized Board of the Council of Forensic Medicine of Türkiye between 2019 and 2023. Demographic, clinical, pathological, and forensic data were evaluated comprehensively.

**RESULTS:**

Most cases were male (79.1%), with a mean age of 57.3±18.5 years. The most common primary cancer was lung (30.7%). Diagnosis relied mainly on medical records (84%), while 12.3% were diagnosed solely by autopsy. Comorbidities were frequent (74.2%), the metastasis rate was high (46.0%), and deaths mostly occurred in hospitals (78.5%). Malpractice claims were present in 36.8%, particularly higher in interventional treatments.

**CONCLUSION:**

Autopsy and histopathological examinations remain essential for diagnostic accuracy in forensic cancer-related deaths. Malpractice allegations were more frequent among cases undergoing surgery and chemotherapy/radiotherapy, although these findings reflect allegations rather than confirmed malpractice. A multidisciplinary approach integrating clinical, pathological, and forensic data is crucial for medicolegal evaluation.

## INTRODUCTION

Cancer remains one of the leading causes of morbidity and mortality worldwide. Current data indicate an increasing cancer burden both globally and in Türkiye. According to GLOBOCAN (Global Cancer Observatory: Cancer Today) 2022 data, millions of new cancer cases and related deaths are reported worldwide, and cancer ranks among the leading causes of death in Türkiye^1,2,3^.

The forensic medical evaluation of cancer cases is particularly important in cases involving sudden and unexpected deaths, allegations of negligence or malpractice related to diagnostic and therapeutic processes, and situations requiring clarification of the cause of death. Findings on malignancy detection in forensic autopsy series vary in the literature, mainly due to differences in study design, autopsy indications, and histopathological examination approaches^4,5^. Therefore, standardized autopsy protocols and detailed histopathological examinations are critical for accurate judicial processes and reliable public health data^6^.

In forensic practice, cancer cases are classified into three groups: primary malignancies presenting as sudden deaths diagnosed at autopsy; suspicious deaths in individuals with known cancer; and cases involving malpractice allegations. Each requires specific evaluation and a multidisciplinary approach^7^.

This study aimed to retrospectively analyze demographic, clinical, and pathological findings, as well as forensic evaluation processes, in cancer-related deaths over five years in Türkiye, and to contribute to forensic practice.

## METHODS

### Case selection and data collection

This retrospective study included 163 forensic cases evaluated by the First Specialized Board of the Council of Forensic Medicine of the Republic of Türkiye between January 1, 2019, and December 31, 2023, in which the cause of death was determined as cancer. Because cases were included according to the date of forensic board evaluation rather than the date of death, some deaths had occurred in earlier years. Cases were selected according to definitive causes of death established by the board after evaluation of forensic files, while those referred for insurance assessment were excluded. Cases referred solely for insurance assessment were excluded because they follow a distinct legal evaluation pathway and were not considered comparable to medicolegal death investigations.

Cases were analyzed in terms of demographic characteristics (sex, age), place and year of death, geographic distribution, hospitalization history, and the interval between the last hospital admission and death. Primary cancer type, diagnostic methods (medical records, statements, autopsy findings), treatment modalities, comorbid systemic diseases, and autopsy findings were also evaluated. Histopathological results, including tumor presence, involved organs and systems, and pathological diagnoses, were recorded. Additionally, metastasis status and localization, toxicological findings, definitive causes of death, and malpractice allegations were assessed. Toxicological categories were based on all substances detected in biological samples. Consequently, chronic disease medications and cardiopulmonary resuscitation drugs could be recorded concurrently within the same case. All variables were analyzed holistically by integrating clinical, pathological, and forensic data. For cause-of-death classification, cases categorized as “cancer and infection/multiple organ failure” were those in which infection or multiple organ failure was identified as the immediate terminal event, whereas “primary cancer and complications” referred to progressive cancer-related mortality without a dominant infectious process.

Histopathological data included both postmortem tissue examinations and available antemortem pathology reports (biopsy and surgical pathology specimens) contained within the forensic case files. Therefore, the number of cases with histopathological information may slightly exceed the number of autopsied cases.

### Data analysis

Data were analyzed using IBM SPSS Statistics v.27. Descriptive statistics were presented as number (n) and percentage (%) for categorical variables, and mean±standard deviation and median (minimum–maximum) for continuous variables. Chi-square and Fisher’s exact tests were used for categorical comparisons; distribution was assessed by the Kolmogorov-Smirnov test. Student’s t-test or Mann-Whitney U test was applied, and Kruskal-Wallis was used for multiple groups. Statistical significance was set at p<0.05. Because the study was exploratory in nature, reported p-values were not adjusted for multiple comparisons and should be interpreted cautiously.

### Ethics approval

Approval for this study was obtained from the Education and Scientific Research Commission of the Council of Forensic Medicine (decision dated April 30, 2024; No. 21589509/2024/323), and the principles of the Declaration of Helsinki were observed.

## RESULTS

Of the 163 cases, 79.1% (n=129) were male. Cases were concentrated in older age groups, most frequently in the 60–69 range (28.2%; n=46). The mean age was 57.26±18.5 years. Most deaths occurred in hospitals (78.5%; n=128), with increased cases between 2020 and 2022. Autopsy was performed in 81.6% (n=133), and 71.8% (n=117) had prior hospitalization. The interval between last admission and death was most often 8–30 days (25.8%; n=42) (Table 1).

**Table 1:** Demographic and clinical characteristics of the cases.

Characteristics		n	%
Sex	Male	129	79.1
	Female	34	20.9
Age groups (decades)	0–9	7	4.3
	10–19	0	0.0
	20–29	7	4.3
	30–39	8	4.9
	40–49	16	9.8
	50–59	39	23.9
	60–69	46	28.2
	70–79	26	16.0
	80–89	12	7.4
	90–99	2	1.2
Place of death	Hospital	128	78.5
	Home	33	20.3
	Nursing home/care facility	2	1.2
Year of death	2016	2	1.2
	2017	6	3.7
	2018	20	12.3
	2019	22	13.5
	2020	28	17.2
	2021	31	19.0
	2022	37	22.7
	2023	17	10.4
Geographical regions	Marmara	59	36.2
	Aegean	17	10.4
	Mediterranean	14	8.6
	Central anatolia	41	25.2
	Black sea	12	7.4
	Eastern anatolia	6	3.7
	Southeastern anatolia	14	8.6
Autopsy status	Performed	133	81.6
	Not performed	28	17.2
	Exhumation	2	1.2
History of hospitalization	Present	117	71.8
	Absent	46	28.2
Time between last hospital admission and death	0–24 h	40	24.5
	2–3 days	20	12.3
	4–7 days	19	11.7
	8–30 days	42	25.8
	1–4 months	12	7.4
	Non-hospital deaths	30	18.4

n: number; %: percentage. Cases were included according to the date of forensic board evaluation (2019–2023); therefore, the year of death may precede the study evaluation period.

The most common primary cancer was lung (30.7%; n=50), followed by small and large intestine (9.2%; n=15), liver (7.4%; n=12), and hematological malignancies (6.7%; n=11); breast cancer accounted for 3.1% (n=5). Diagnosis relied mainly on medical records (84%; n=137), followed by statements (70.6%; n=115) and autopsy findings (48.5%; n=79). In 12.3% (n=20), diagnosis was established solely by autopsy.

Medical therapy alone was most common (26.4%; n=43), with combined treatments also frequent (Table 2). At least one systemic disease was present in 74.2%. Cardiovascular diseases (52.2%; n=85) were most frequent, followed by diabetes mellitus (18.4%; n=30) and chronic obstructive pulmonary disease (COPD) (17.2%; n=28). No or unknown comorbidity was present in 25.8% (n=42). At autopsy, mass lesions and serous fluid accumulation (39.3%; n=64) predominated; no macroscopic findings were observed in 12.3% (n=20), and prior organ removal in 4.9% (n=8).

**Table 2:** Clinical, pathological, toxicological characteristics and outcomes of the cases.

Characteristics		n	%
Treatment modalities	Medical treatment only	43	26.4
	Unspecified/unknown	32	19.6
	Surgery+CT/RT+medical	27	16.6
	CT/RT+medical	25	15.3
	Surgery+medical	13	8.0
	Surgery only	12	7.4
	CT/RT only	11	6.7
Systems with histopathologically detected tumor	Respiratory	31	19.0
	Gastrointestinal	25	15.3
	Hematolymphoid	5	3.1
	Genitourinary	7	4.3
	Central nervous system	2	1.2
	Endocrine	1	0.6
	Other	5	3.1
	No tumor detected	87	53.4
Toxicological findings	CDD+CPR drugs	76	46.6
	CDD	32	19.6
	CPR drugs	6	3.7
	Substances of abuse+CPR drugs+CDD	4	2.5
	Chemotherapeutic agents	2	1.2
	No drug/substance detected	19	11.7
	Unknown	24	14.7
Definitive cause of death	Primary cancer and complications	86	52.8
	Cancer and infection/MOF	45	27.6
	Cancer and systemic diseases	12	7.4
	PCSC	9	5.5
	Other (e.g., trauma and cancer)	8	4.9
	UA (possible cancer)	2	1.2
	Drug intoxication and cancer	1	0.6
Malpractice allegation	Present	60	36.8
	Absent	103	63.2

CT: chemotherapy; RT: radiotherapy; CDD: chronic disease drugs; CPR: cardiopulmonary resuscitation; MOF: multiple organ failure; PCSC: primary cancer surgery and complications; UA: undetermined at autopsy; n: number; %: percentage.

Histopathological examination was performed in 82.8% (n=135), with tumors detected in 46.6% (n=76). The lung was most frequently involved (17.8%; n=29), followed by liver (6.1%; n=10), bone marrow (3.7%; n=6), and pancreas (3.1%; n=5). The respiratory system predominated (19%; n=31) (Table 2). The most common diagnosis was malignant tumor (24.5%; n=40), followed by adenocarcinoma (7.4%; n=12) and squamous cell carcinoma (3.7%; n=6).

Metastasis was identified in 46.0% (n=75), most commonly in the liver (22.1%; n=36), followed by brain (10.4%; n=17) and heart (9.2%; n=15). Toxicology most frequently showed combined chronic disease and resuscitation drugs (46.6%; n=76). The most common cause of death was primary cancer and its complications (52.8%; n=86), and malpractice allegations were present in 36.8% (n=60) (Table 2).

Lung cancer was more frequent in males (32.6%; n=42) than females (23.5%; n=8), with a significant association between cancer type and sex (p=0.016), but not age (p=0.196). Respiratory system cancers were more frequent in those ≥60 years (33.1%; n=54), without significance (p=0.106). No associations were found between systems and metastasis (p=0.260) or causes of death (p=0.303). Medical therapy was more frequent in cases without histopathologically detected tumors (75.9%; n=66). Surgical treatment was significantly associated with histopathological tumor detection (p=0.014), whereas no significant associations were observed for chemotherapy/radiotherapy (p=0.093) or medical treatment (p=0.139). Malpractice allegations were more frequent among cases undergoing surgery (48.1%; n=25) and chemotherapy/radiotherapy (47.6%; n=30) than among those who did not receive these treatments (p=0.041 and p=0.023, respectively), whereas no significant association was observed for medical treatment alone (p=0.265).

## DISCUSSION

This study demonstrates a clear relationship between clinical processes and forensic medical evaluation in cancer-related deaths, indicating a multidimensional interaction between clinical, pathological, and forensic components.

Most cases were male (79.1%) and in older age groups, with a mean age of 57.26±18.5 years. Similarly, Çengel et al.^8^ reported a male proportion of 79.7% in malignancy cases. This male predominance is consistent with cancer epidemiology, as GLOBOCAN 2022 data show higher incidence rates in men both globally and in Türkiye^9^. These findings highlight the importance of demographic characteristics in forensic evaluation.

The predominance of hospital deaths and limited nursing home deaths suggests insufficient institutional care for terminal cancer patients. This aligns with reports indicating limited institutional care capacity in Türkiye^10^ and high care needs in nursing home populations^11^. The increase in cases during 2020–2022 may reflect disruptions in cancer care during the COVID-19 pandemic, including delays in diagnosis and treatment^12^, potentially leading to advanced-stage disease and increased forensic case burden. These findings underscore the need to strengthen palliative and end-of-life care services.

The high rate of hospitalization and the concentration of deaths within early and intermediate periods after admission suggest the importance of clinical complications. The coexistence of cancer and infection, along with the predominance of respiratory system cancers, supports the role of infections in mortality. Medical intervention-related complications, particularly lower respiratory tract infections, are more frequent in hospital deaths^13^. Thus, mortality in cancer patients is influenced not only by disease progression but also by complications arising during clinical care, emphasizing the need for comprehensive evaluation of healthcare history.

Within this forensic referral cohort, lung cancer was the most frequently identified primary malignancy, and respiratory and gastrointestinal system tumors predominated. The higher frequency of lung cancer among men was broadly consistent with epidemiological observations reported in the literature^9^. However, because the study population consisted exclusively of cases referred for medicolegal evaluation, these findings should not be interpreted as representative of the overall distribution of cancer-related deaths in Türkiye. The relatively low frequency of breast cancer among women may reflect the forensic characteristics of the study population. Histopathological findings, with the lung as the most frequently involved organ and the respiratory system predominating, support this distribution. The absence of tumor findings in more than half of cases may be related to widespread metastasis and organ failure in terminal stages. Diagnosis based mainly on medical records, with a notable proportion established only at autopsy, highlights the limitations of clinical evaluation and the importance of autopsy and histopathology^14,15^. These findings emphasize the necessity of integrated evaluation for diagnostic accuracy.

Multimodal treatment approaches are emphasized in oncology, with therapy guided by tumor characteristics^16^. In this study, medical therapy was most common, while combined treatments were also frequent. The significant association between surgical treatment and histopathological diagnosis indicates concordance between treatment and diagnostic processes. However, missing treatment data in 19.6% of cases underscores the importance of complete medical documentation, as incomplete records may negatively affect forensic processes^17^. Systematic documentation is therefore critical in both clinical and forensic contexts.

Comorbid diseases are common in cancer and significantly influence prognosis^18,19^. In this study, at least one systemic disease was present in approximately three-quarters of cases, consistent with the literature. The higher rate compared to the 48% reported by Karakoç^20^ may be due to inclusion of only fatal cases. Cardiovascular diseases and diabetes mellitus were the most common comorbidities, consistent with Renzi et al.^18^, while respiratory diseases such as COPD support their negative impact, particularly in lung cancer cases^19^.

Macroscopic examination is a fundamental step in autopsy diagnosis, although findings may be limited, requiring histopathological confirmation^21^. The detection of mass lesions and serous fluid accumulation, alongside cases without macroscopic findings, supports this. Prior surgical removal of organs may limit macroscopic evaluation, necessitating correlation with clinical history. The predominance of malignant tumors, adenocarcinoma, and squamous cell carcinoma aligns with the literature, particularly for lung cancer subtypes^22^. The most frequent metastasis sites—liver, brain, and heart—are consistent with organotropic spread patterns^23^. The relatively high frequency of cardiac metastases should be interpreted cautiously. Cardiac metastases are often underdiagnosed during life and may be detected more frequently during postmortem examinations, particularly in advanced-stage disease. However, the lack of significant association between systems and metastasis suggests a multifactorial process. These findings reinforce the importance of integrating macroscopic, histopathological, and clinical data.

In this study, toxicological analysis most frequently revealed the combined presence of medications for chronic diseases and cardiopulmonary resuscitation drugs. Additionally, the low detection rate of chemotherapeutic agents and the absence of any detectable substances or unavailable results in some cases are noteworthy. In forensic toxicology, the analysis of biological samples is essential for identifying drugs used prior to death and evaluating their potential contribution to mortality^24^. This may be related to the postmortem stability of chemotherapeutic agents, analytical limitations, and variability associated with postmortem processes. Therefore, toxicological findings should be interpreted within the clinical and forensic context of the case.

Malpractice allegations were identified in 36.8% of cases and were significantly more frequent in those undergoing surgery and chemotherapy/radiotherapy. In oncology, malpractice claims are often associated with treatment processes and care organization^25^. However, this study evaluated malpractice allegations rather than confirmed malpractice determinations. The higher frequency of allegations among cases receiving surgery or chemotherapy/radiotherapy may reflect treatment complexity, disease severity, or patient and family expectations rather than deficiencies in care. Although complications may lead to serious outcomes, not all adverse events constitute malpractice. In fatal cases, autopsy findings remain critical for the evaluation of malpractice claims.

### Limitations

This study has several limitations. First, the study population consisted exclusively of cases referred to the First Specialized Board of the Council of Forensic Medicine, introducing referral and selection bias and limiting the generalizability of the findings. Therefore, the observed distributions of cancer types, clinical characteristics, and medicolegal findings may not reflect those of all cancer-related deaths in Türkiye. Second, treatment information was unavailable in 19.6% of cases. Third, no non-forensic comparison group was available. Fourth, the study evaluated malpractice allegations rather than substantiated or legally confirmed malpractice determinations. Fifth, toxicological findings were interpreted qualitatively because quantitative postmortem drug concentrations were not consistently available. Finally, although additional multivariable analyses may have provided further insights, the retrospective nature of the study and the lack of access to an analyzable individual-level dataset precluded further modeling beyond the predefined analyses.

## CONCLUSION

This study demonstrated that autopsy and histopathological examinations remain essential for diagnostic accuracy in forensic cancer-related deaths. Malpractice allegations were more frequent among cases undergoing surgery and chemotherapy/radiotherapy, although these findings reflect allegations rather than confirmed malpractice. The results highlight the importance of integrating clinical, pathological, and forensic data through a multidisciplinary approach in medicolegal evaluations of cancer-related deaths.

## Data Availability

The datasets generated and/or analyzed during the current study are available from the corresponding author upon reasonable request.
